# Increased Extinction Potential of Insular Fish Populations with Reduced Life History Variation and Low Genetic Diversity

**DOI:** 10.1371/journal.pone.0113139

**Published:** 2014-11-19

**Authors:** Michael Hellmair, Andrew P. Kinziger

**Affiliations:** 1 Department of Fisheries Biology, Humboldt State University, Arcata, California, United States of America; 2 FISHBIO, Chico, California, United States of America; Swansea University, United Kingdom

## Abstract

Theoretical work has shown that reduced phenotypic heterogeneity leads to population instability and can increase extinction potential, yet few examples exist of natural populations that illustrate how varying levels expressed diversity may influence population persistence, particularly during periods of stochastic environmental fluctuation. In this study, we assess levels of expressed variation and genetic diversity among demographically independent populations of tidewater goby (*Eucyclogobius newberryi*), show that reductions in both factors typically coincide, and describe how low levels of diversity contribute to the extinction risk of these isolated populations. We illustrate that, for this annual species, continuous reproduction is a safeguard against reproductive failure by any one population segment, as natural, stochastically driven salinity increases frequently result in high mortality among juvenile individuals. Several study populations deviated from the natural pattern of year-round reproduction typical for the species, rendering those with severely truncated reproductive periods vulnerable to extinction in the event of environmental fluctuation. In contrast, demographically diverse populations are more likely to persist through such periods through the continuous presence of adults with broader physiological tolerance to abrupt salinity changes. Notably, we found a significant correlation between genetic diversity and demographic variation in the study populations, which could be the result of population stressors that restrict both of these diversity measures simultaneously, or suggestive of a causative relationship between these population characteristics. These findings demonstrate the importance of biocomplexity at the population level, and assert that the maintenance of diversity contributes to population resilience and conservation of this endangered species.

## Introduction

Life-history diversity and genetic variation are two forms of biodiversity deemed worthy of conservation by the World Conservation Union (IUCN), and are considered essential to retaining fitness and long-term adaptive potential of populations [1]–[3]. Further, it is often invoked that expressed forms of diversity contribute to the stability of biological systems: high species diversity often stabilizes ecosystem processes [4]–[7], and variation among populations of individual species buffers fluctuations in the species’ cumulative abundance and the ecosystem services it provides [8]–[11]. However, the role of diversity within individual populations of a species has remained largely unexplored, though there is some experimental evidence that post-disturbance variance in abundance is stabilized by diversity, and extinction risk becomes more predictable as a consequence [12].

In this study, we investigated age composition and genetic diversity among isolated populations of tidewater goby (*Eucyclogobius newberryi*), a short-lived, endangered fish that inhabits dynamic coastal habitats subject to frequent environmental changes [13]–[15]. We discuss the importance of life history variability *within* geographically isolated populations in the context of the species’ biology, and suggest that reduced levels of variation, particularly in the temporal extent of the reproductive period, render affected populations more susceptible to localized extirpation during periods of environmental disturbance.

**Figure 1:**
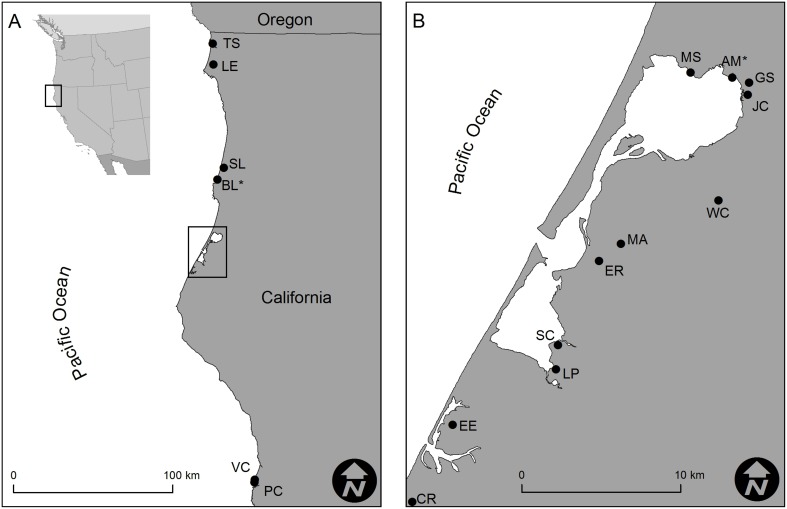
Map depicting locations of tidewater goby populations on the northern California coast. Panel A shows populations directly adjacent to the Pacific Ocean, including Tillas Slough (TS), Lake Earl (LE), Stone Lagoon (SL), Big Lagoon (BL*), Virgin Creek (VC) and Pudding Creek (PC). (B) McDaniel Slough (MS), Arcata Marsh (AM*), Gannon Slough (GS), Jacoby Creek (JC). Panel B illustrates isolated populations found around the margin of Humboldt Bay and the Eel River estuary, including Wood Creek (WC), Elk River (ER), Martin Slough (MA), Long Pond (LP), Salmon Creek (SC), Connick Ranch (CR) and Eel River (EE). Populations noted with an asterisk represent focal populations for otolith age analysis.

The tidewater goby is a small (<60 mm total length), annual fish endemic to California, that occurs in brackish, isolated, and often very small (less than 10 hectares) estuaries along the coast, from just south of the Oregon border to San Diego, USA [13]–[15]. Among insular populations of tidewater goby, levels of genetic differentiation are often very high, despite geographic proximity [16]. Further, the level of genetic diversity within populations varies widely (as measured by allelic richness and heterozygosity; see [16] for a discussion on population structure of the species’ northern range; Figure 1; Table 1). Migration between populations is unlikely, as suitable habitats are typically separated from the Pacific Ocean by sandbars most of the year [13], [14], [16], [17], and requires coincident breaching events between sites. Breaching generally occurs 1–2 times annually following periods of high freshwater input and large surf, but may not happen for several successive years [18]. These stochastic events commonly cause rapid draining of the estuary and influx of ocean water over subsequent tidal cycles. Breaching events are physiologically challenging for fishes due to rapid changes in water level, temperature and salinity. Abrupt changes in salinity in particular are known to be lethal to many fish species, including gobies, especially during early life history stages [19]–[21].

**Table 1:** Habitat size, location, demographic parameters and genetic diversity measures (across nine microsatellite loci) of 17 tidewater goby populations in northern California.

Population	Coordinates		Area (hectares)	*n*	Mean size^a^ (mm)	SD	Size range^a^ (mm)	*H*_E_ b	*A_r_* c
	N	W							
Virgin Creek (VC)	39°28′11.29″	123°48′10.49″	4.5	60	28.85	3.84	18	0.57^d^	3.69
Big Lagoon (BL)	41°09′51.48″	124°07′50.16″	612.5	60	32.47	6.46	25	0.56^d^	4.92
Stone Lagoon (SL)	41°13′58.44″	124°05′03.48″	236.7	60	28.95	8.39	32	0.52^d^	4.89
Pudding Creek (PC)	39°27′10.81″	123°48′18.62″	9.5	60	35.28	5.16	20	0.45^d^	2.94
Eel River (EE)	40°39′09.45″	124°17′34.77″	108.5	60	40.78	4.38	20	0.28^d^	2.67
Elk River (ER)	40°44′51.17″	124°11′14.20″	35.1	60	32.93	6.55	27	0.28^d^	1.83
Lake Earl (LE)	41°48′59.18″	124°10′54.45″	1085.4	60	22.10	3.86	17	0.26	3.18
Tillas Slough (TS)	41°55′59.90″	124°11′25.74″	6.6	60	25.72	2.00	8	0.26	2.40
Connick Ranch (CR)	40°36′30.36″	124°19′18.02″	6.4	60	41.40	4.35	17	0.25	2.48
Long Pond (LP)	40°41′08.45″	124°13′02.22″	2.3	60	38.1	4.05	17	0.24	2.20
Salmon Creek (SC)	40°41′57.72″	124°12′58.71″	396.9	60	28.90	4.21	18	0.23^d^	2.21
Gannon Slough (GS)	40°51′02.31″	124°04′43.17″	18.2	60	41.15	2.79	13	0.22^d^	1.87
Martin Slough (MA)	40°45′27.18″	124°10′16.93″	0.2	60	30.22	3.30	14	0.21	1.78
McDaniel Slough (MS)	40°51′20.14″	124°07′20.89″	34.8	31	27.23	2.65	12	0.18^d^	1.75
Jacoby Creek (JC)	40°50′37.59″	124°04′46.21″	6.2	58	20.00	2.54	11	0.16^d^	1.66
Wood Creek (WC)	40°47′00.95″	124°05′57.38″	0.4	59	31.65	3.75	13	0.10^d^	1.36
Arcata Marsh (AM)	40°51′11.86″	124°05′28.71″	0.2	165	40.69	1.98	11	0.08	1.36

a Size is defined as total length (TL), and size range the difference between the largest and smallest individual from *n* individuals sampled.

b Mean expected Hardy-Weinberg heterozygosity.

c Rarefied allelic richness.

d Heterozygosity estimates obtained from McCraney et al. (2010).

A number of life history adaptations allow tidewater goby populations to persist in these environmentally challenging habitats. Most notably, reproduction occurs throughout the year, though increased spawning activity is generally observed during summer and fall [13], [14], [22], [23]. Individuals can become reproductively mature at lengths under 30 mm, and can actively spawn over prolonged time periods (several months) [22], [23]. As a result, tidewater goby populations are typically characterized by a broad size and age structure through the continuous presence of both juvenile and adult individuals. This reproductive strategy may be considered a bet-hedging trait, an adaptation to unpredictable, though frequent, environmental variance in the habitat of this species that maximizes reproductive output during time periods that are generally favorable (summer and fall, when the likelihood of breaching is lowest) [18], [24], yet safeguards populations from extirpation through the continuous presence of resilient adult individuals in the event of stochastic reproductive failure by any one population segment.

In this study, (1) we used a controlled experiment to confirm that salinity increases associated with breaching causes mortality to the early life stages, and (2) show how continuous reproduction (as measured by population age composition) can buffer isolated populations of tidewater goby against adverse impacts (high mortality) resulting from these events. Further, (3) we assessed the genetic diversity of 17 populations of this species in northern California, and (4) investigated relationship between genetic diversity measures and demographic heterogeneity after accounting for habitat size (as a proxy of habitat heterogeneity). We discuss the results in the context of the biology of this endangered fish species, and argue that resilience to environmental stochasticity increases when life history variability and healthy levels of genetic variation are retained in a population.

## Materials and Methods

### Ethics statement

All work was conducted under California Scientific Collecting Permit SC-10527 and in concordance with institutional protocol at Humboldt State University (Institutional Animal Care and Use Committee 08/09.F.44.A & 10/11.F.78-C), where all samples have been deposited in the scientific fish collection (HSU Catalog #4890-4913).

### Salinity trials

To evaluate size-specific mortality associated with salinity increases experienced by tidewater goby populations during a breaching event, we exposed two size groups of tidewater goby (small and large) captured in BL (14–35 mm TL: 11 replicates, 10 individuals each; 36–55 mm TL: 5 replicates, 10 individuals each; Figure 1) to a 20‰ increase in salinity over ambient lagoon salinity (we mixed water from the adjacent Pacific Ocean [35‰] with lagoon water to bring the salinity to 26‰). Though the magnitude of salinity increases during natural breaching events are highly variable, depending on tidal phase, surf levels and freshwater input, salinity levels approaching that of seawater have been regularly observed. We considered an intermediate increase to 26‰ sufficient to identify breaching events as a cause of mortality and evaluate differences in size-specific survival. Two control groups (5 fish 31–55 mm TL, 16 fish 14–30 mm TL) were kept in lagoon water. These groups were not intended for inclusion in statistical analysis, but to indicate shortcomings of the experimental design in case of mortality to those individuals. All replicates were held in 10 liter tanks, aerated consistently and monitored for 16 hours. To limit the number of overall mortalities, we discontinued the experiment after only 16 hours as clear differences in survivorship between the groups had become apparent.

Sample size for this experiment was limited by numbers permitted for research take under collecting permits of this protected fish and subject to capture success in the wild. Following the observation period, we classified all individuals as dead or alive and measured them to the nearest millimeter (TL). We used Welch’s two sample T-test to test for significant differences in survival between size groups.

### Population age structure and genetic diversity

We captured tidewater goby using a 3.3 m by 1.3 m seine with a 1.58 mm mesh in water no deeper than the height of the seine and no shallower than 0.2 m. As neither our field observation nor any literature report indicate age- or size-specific segregation of tidewater goby, we considered collections representative subsamples of the respective goby populations.

We obtained estimates of genetic diversity and corresponding individual length measurements (total length, TL, in millimeters, mm) for eleven populations from a previous study (sampled in 2006) [16], and non-lethally collected length measurements and genetic tissue samples (fin clips) for six additional populations (in 2011), to compile a dataset representing 17 populations of tidewater goby in northern California (Table 1; Figure 1). We estimated population genetic diversity for the six additional populations using the identical set of nine microsatellite loci developed specifically for tidewater goby and according to methods outlined in detail in the literature [16], [25], [26]. We estimated rarified allelic richness using the program HP-RARE [27] to control for the correlation between observed allelic diversity and sample size [28]. Alleles were rarified to a sample size of 31, the smallest sample size of our population groups.

In organisms with indeterminate growth, particularly those with a short lifespan, the size distribution of individuals is conservatively reflective of the population’s age composition. In other words, while individuals of the same age may obtain different sizes at time *t* (therefore inflating the measure of actual temporal spread), it is improbable that many individuals of different ages reach identical, or similar, sizes at time *t* (deflating temporal spread). Following this reasoning, we consider the length range of samples an approximation of the variation in ages within populations of this short-lived fish. However, to further validate that size measurements provide a nonlethal alternative to direct (lethal) age estimation, we conducted a more detailed age and growth study on two large populations using otolith analysis (described in the following section).

We evaluated the relationship between the length ranges observed in our study populations (difference, in mm, between the smallest and largest individual) and their genetic diversity (mean Hardy-Weinberg expected heterozygosity [*H*_E_] and allelic richness [*A_r_*]) through linear regression analysis. Alternatively, we used the standard deviation (SD) in lengths observed within populations to investigate the relationship between demographic variation and genetic diversity, as the observed length range may be strongly affected by presence of rare individuals with atypical (extreme) sizes.

As the size of the habitat to which a population is constrained may influence the duration of the reproductive period and limit populations size (at any time, larger habitats have a broader range of depths, temperatures and other environmental variation that may facilitate prolonged reproductive periods), we used area (log_10_ *hectares*) to control for effects of environmental differences among the isolated habitats in a general linear model fit.

### Additional sample collection, age determination and estimation of growth parameters

Unlike most animal taxa, fishes can often be aged directly through analysis of periodically deposited increments in bony structures, most notably their otoliths (earstones). We used otolith microstructural analysis to estimate the ages (in days) for large numbers of individuals from two populations in northern California, USA, for age determination (Big Lagoon [BL] and Arcata Marsh [AM], *n* = 413 and 88, respectively; Figure 1). Due to concerns regarding periodic lethal sampling of this endangered species, we chose two populations with high abundance (several thousand individuals) and considered representative of stocks with high and low genetic diversity (*H*_E_ = 0.56 and 0.08, *A_r_* = 4.92 and 1.36, respectively) for comparison. In Big Lagoon (BL), we collected individuals from April 2009 until August 2010, and sampled the second location, Arcata Marsh (AM), from April 2009 until October 2011. We measured all individuals in the field to the nearest millimeter (total length, TL), and sacrificed and preserved a subsample of 20–25 fish spanning the length range of goby encountered (by subjecting them to an overdose of MS222, Tricaine methanesulfonate; preservative: 90% ethanol). Concurrent with field sample collection, we obtained salinity measurements (parts per thousand, ‰) using a VEE GEE STX-3 refractometer.

We extracted the sagittal otoliths, rinsed them in water and ethanol and mounted them on a microscope slide using wet’n’wild “Wild Shine” Clear Nail Protector (type 401A). After curing, we polished the otoliths by hand using waterproof sandpaper (GatorGrit, type 1500-b) and 6 µm lapping film (Allied High Tech Products, Inc., Diamond Lapping Film #50-30265) to reveal daily increments. We counted daily increments under a compound microscope under 500X total magnification, using an oil-immersion lens (MEIJI Model #10824), and captured images captured with a Lum*e*nera Infinity 1 camera (ImagePro Plus software, version 7.0). We began enumeration of daily increments at the first continuous increment outside the core region, and counted increments continuously to the otolith margin. We discarded the sample when continuous enumeration not possible on either of the two otoliths.

To validate daily growth increment deposition, we induced chemical marks in otoliths by immersing tidewater goby in a 5% calcein solution. We marked a total of 1441 tidewater goby and released them into their natal environment (BL, Figure 1), and used an SE-MARK detector (Western Chemical, Co.) to identify marked individuals during recapture events five, ten, twenty and twenty-eight days after marking. We retained five marked individuals during each recapture event, prepared their sagittal otoliths as indicated above and examined them under a fluorescence microscope. We estimated annual survivorship for each population using daily age estimates [29] and fit a growth model [30] to daily length at age estimates for each population (n = 413 and 88 for BL and AM, respectively):
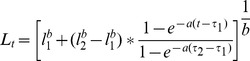
where 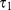 and 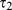 are the ages of the youngest and oldest fish in the sample (see results), 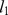 and 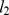 the corresponding lengths at that age, *t* is age (in days), *a* is the inverse of time, and *b* is a dimensionless constant. We then reconstructed birthdate distributions for these two populations by tabulating, for each fish, the estimated daily age subtracted from its collection date.

## Results

### Salinity trials

**Figure 2:**
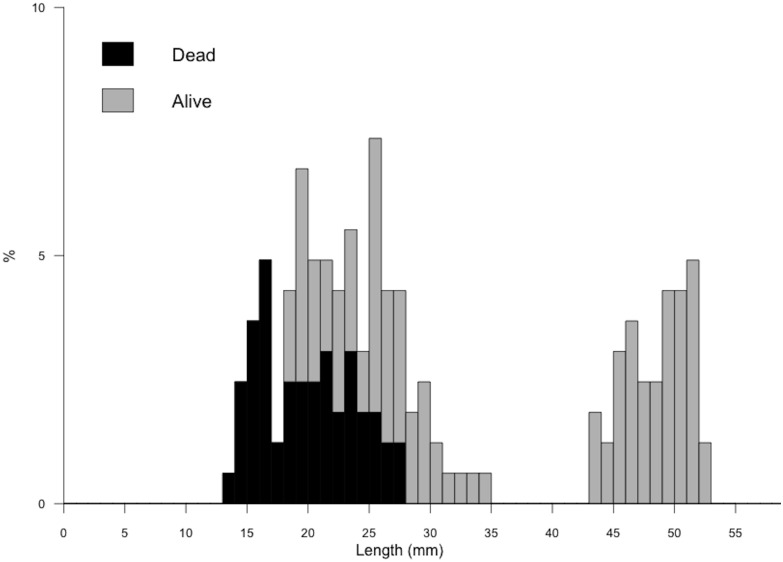
Size-specific mortality of tidewater goby (total n = 163) subjected to a 20‰ increase in salinity for 16 hours, illustrating high mortality of juvenile stages and resilience of adults. Mean mortality across all 11 replicates of small individuals (14–35 mm) was 50.4%, and no mortality occurred in any replicates of adult fish (43–53 mm) or the control groups.

Mean mortality across 11 replicates of the “small” category was 0.50 (SD = 0.10), and 0.0 across the 5 replicates of the “large” category (Figure 2), illustrating that the chance of surviving a salinity increase is significantly lower for small (young) fish (Welch’s 2-sample t-test; *p*<0.001). No mortality was observed in control groups of either size class. Surviving fish were released into their natal environment.

### Population age structure and genetic diversity

Length-frequency distributions were highly variable among populations, with size ranges and mean sizes ranging from 8 mm to 32 mm and 20.0 to 41.4 mm, respectively. Considering that the maximum size of tidewater goby encountered during our surveys was 55 mm TL, these differences among population are substantial. Populations separated by a distance of less than four kilometers and collected within a one-week time span exhibited different mean lengths (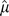 = 20, 27.23 and 41.15 mm; *p*<0.001 ANOVA, Table 1), corresponding to mean ages of 42, 107, and 341 days, respectively (using growth parameters estimated from length at age data for the nearby AM population) (Table 1, Table 2, Figure S1). Monthly collections from BL over a period of 18 months confirm that an observed broad size range is not a seasonal occurrence, but a pattern that can be observed any month of the year (Figure S2).

**Table 2:** Estimates of growth parameters (Schnute 1981) for two northern California populations of tidewater goby, where *n* is the number of samples, 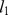 and 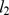 are the lengths of the youngest and oldest fish in the sample, *a* is the inverse of time, and *b* is a dimensionless constant.

	Big Lagoon	Arcata Marsh
*n*	413	88
a (SE)	−0.004(0.002)	−0.007(0.006)
b (SE)*	2.50(0.62)	5.53(2.33)
*l*_1_ (SE)	12.85(0.96)	15.00(2.41)
*l*_2_ (SE)*	55.94(1.24)	42.40(0.86)

Genetic diversity was also highly variable among populations, and expected heterozygosity (*H*_E_) ranged from 0.08 to 0.57, while rarefied allelic richness (*A_r_*) ranged from 1.36 to 4.92 (Table 1). No loci or populations deviated significantly from Hardy-Weinberg Equilibrium (genotypes of individuals included in this study can be found in File S1).

**Figure 3:**
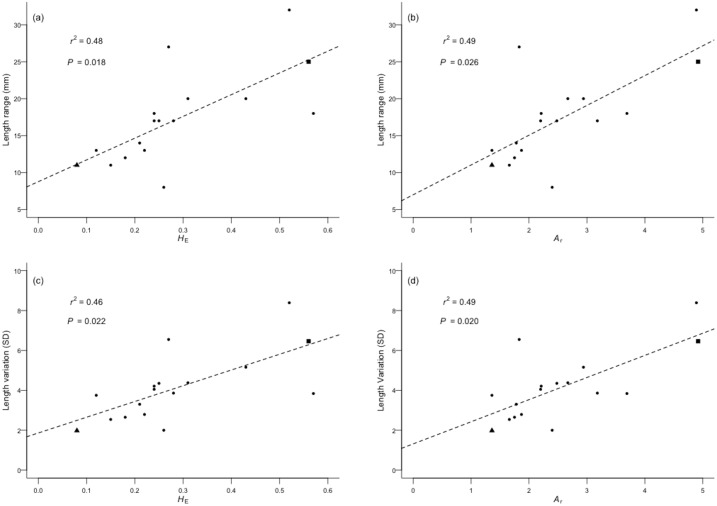
Relationship between observed length range (mm) and (a) mean Hardy-Weinberg expected heterozygosity (*H*_E_), (b) rarefied allelic richness (*A_r_*) across nine microsatellite loci among all known tidewater goby populations on the northern California coast. Populations indicated with a triangle and square indicate focal populations with low and high *H*_E_, respectively, for otolith microstructural analysis.

We detected a correlation between the temporal extent of the reproductive season and genetic diversity. A significant proportion of the variation in demography (as measured by observed size ranges and SD) was explained by expected heterozygosity (*p* = 0.018 and 0.022, respectively) after accounting for the effects of habitat area in a general liner model analysis (model: range(mm)/SD = log_10_(hectares)+*H*_E_). Similarly, allelic richness explained a significant proportion of age/size variation (model: range(mm)/SD = log_10_(hectares)+*A_r_*; *p* = 0.026 and 0.020). Though explaining some of the variation, the effect of habitat area was not significant in any of the four models (*p*>0.2), and this variable was excluded from the model fits for illustration purposes. The univariate relationships between these two measures of genetic diversity and two indicators of demographic diversity in goby populations are shown in Figure 3.

### Additional sample collection, age determination and estimation of growth parameters

**Figure 4:**
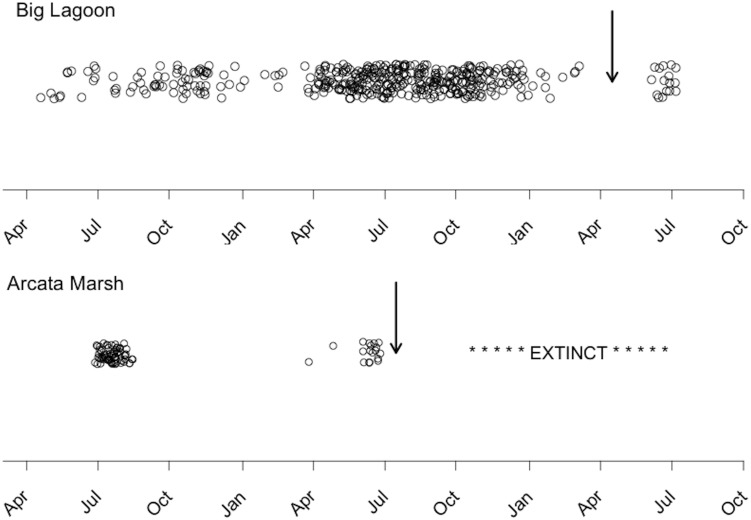
Individual birthdates of tidewater goby estimated from daily otolith increment counts in Big Lagoon (BL), CA, and Arcata Marsh (AM), CA. The Big Lagoon population is characterized by nearly continuous reproduction throughout the year, while the Arcata Marsh population exhibits a severely truncated reproductive period. Salinities in BL and AM ranged from 2‰ to 14‰ and 9‰ to 15‰, respectively, for the duration of the study period, with two exceptions, indicated by vertical arrows (BL: from 13‰ [April 2010] to 27‰ [May 2010]; AM: from 12‰ [July 2009] to 34‰ [August 2009]). High salinities attributed to stochastic salinity increases were lethal to young individuals and resulted in subsequent extinction of the AM population. The timeline illustrated by the horizontal axis begins in April 2008.

The minimum collection goal of 150 individuals per month was met for the BL population every month from April 2009 to August 2010, except June 2009 (n = 28, Figure S2). For the AM population, the collection goal was met from April 2009 to July 2009. In August 2009, extensive sampling yielded only five adult tidewater goby (TL>35 mm), and rigorous sampling using a variety of collection methods the following 24 months suggests that tidewater goby went extinct at this site. Salinities ranged from 2‰ to 14‰ in BL in all months except May 2010 (27‰) following a natural breaching event. In AM, salinity measurements ranged from 9‰ to 12‰ between April and July 2009, increased to 34‰ in August 2009, and decreased to 15‰ in September. Both populations experienced a stochastic salinity increase during the study period. In the AM population with (low genetic diversity, little variation in individual ages/size) no individuals smaller than 30 mm TL (comprising over 94% of the population shortly prior to the salinity increase; Figure S3) were found after this event, and despite extensive repeated surveys over a period of two years, tidewater goby could no longer be documented at this location, indicating localized extinction. The BL population with (high genetic diversity, broad range of ages/sizes) experienced a period of no births associated with the salinity increase (Figure 4), but persisted owing to the presence of large individuals tolerant of abrupt salinity changes.

Counts of increments from the fluorescent mark to the otolith margin matched the number of days between marking and sacrifice for all samples with a detectable calcein mark (ten days: n = 1, 20 days: n = 1, 28 days: n = 2). Daily age estimates ranged from 26 to 363 days for gobies sampled from the AM and from 48 to 421 days for the BL population. Estimates of instantaneous mortality rate of tidewater goby from the BL and AM populations correspond to 2.4% and 1.32% annual survivorship [30], respectively, confirming that few individuals survive longer than one year. Estimated growth curves for tidewater goby from BL and AM are illustrated in Figure S1, and parameter estimates are listed in Table 2.

Back calculation of birth dates for individuals from the AM population indicated a truncated reproductive period that manifests itself as a narrow size distribution of individuals (Figure 4; Table 1). In contrast, nearly year-round reproduction the genetically diverse BL population resulted in the presence of a broad range of individual sizes in this population (Figure 4; Table 1).

## Discussion

The buffering effects of biological diversity on ecosystem processes, likened to the portfolio effect from economic theory, are frequently invoked as an important argument for the conservation of biodiversity [31]–[33]. These principles have been extended to the metapopulation level, illustrating that variability and biocomplexity among fish stocks will increase the resilience of metapopulations [8], [9]. The importance of among-population life history variability to the stability of meta-populations is increasingly recognized and has recently been quantitatively illustrated for a commercially exploited species for which long-term abundance estimates exist [9], [33]. Diversity among populations belonging to a stock complex buffers variability in overall abundance by spreading the risk of stochastic declines over many population segments, increasing the stability of cumulative meta-population abundance. This resilience is of great importance to the temporal stability of commercial exploitation, and improves the viability and sustainability of the stock complex in the long term, similar to the increased likelihood of stable returns attributed to diverse economic portfolios [8], [9], [33].

The results presented herein suggest that this portfolio effect not only applies to species which occur as distinct segments in a stock complex, but also to the resilience of individual, isolated populations. For tidewater goby, continuous reproduction independent of season (and the resulting diverse population age composition) appears to act as a natural safeguard against localized extinction by spreading the risk of high juvenile mortality across many different population segments. The implication of a truncated single spawning period for long-term population persistence was illustrated during a temporary increase in salinity at the Arcata Marsh (AM), a natural yet stochastic process in coastal habitats of tidewater goby. The timing of this disturbance coincided with a period of population turnover, shortly after the brief reproductive period but before juveniles could grow to sufficient size to better tolerate elevated salinity levels. Juvenile individuals, comprising over 94% of the population, vanished as a consequence of this disturbance (Figure S3). As length frequency distributions and results from otolith analysis suggest, the few surviving adults were nearing the maximum of longevity and their emaciated state indicated that they were post-reproductive. Extensive field surveys over the two subsequent years confirmed extinction of this previously large population. Such increased susceptibility of early life history stages, including eggs, to salinity fluctuations is not unusual and has been shown for a number of estuarine goby species [19]–[21]. A controlled experiment confirmed that increases in salinity are lethal to juvenile tidewater goby, supporting salinity change associated with breaching as the cause of extinction.

A similar salinity increase, but at a different location (Big Lagoon, BL), resulted in a stark decrease in abundance of smaller individuals (<35 mm TL, Figure S3) and an absence of new recruits to the population from the time period surrounding the breach (Figure 4). This population persisted, owing to the presence of large numbers of adult fish that can withstand physiological challenges associated with salinity increases. Though the salinity increase experienced by individuals in the Arcata Marsh (34‰) was greater in magnitude than in Big Lagoon (27‰) and during our salinity challenge experiment, adult tidewater goby have been documented in the wild at salinities of up to 42‰ [14]. These findings suggest that naturally occurring salinity increases are a significant population stressor whose effects can be buffered by demographic heterogeneity [34].

It should be emphasized that breaching events are an important mechanism in establishing connectivity among habitats typically subject to physical separation, and thus provide seasonal opportunity for migration and colonization of suitable habitats. As such, this natural process to which tidewater goby are well adapted (through broad physiological tolerances by adult individuals) is an important component of the species’ biology. Consequently, the occurrence of breaching should not cause excessive stress on populations are characterized by a diverse age composition despite high juvenile mortality; rather, breaching threatens population persistence only if the population is composed of individuals of similar age, and if it occurs at a time when the great majority of individuals are of small size (shortly after peak reproduction). Further, the importance of breaching events as a population stressor may differ across the species’ range: previous work suggests that populations in the southern portion of the species’ range are subject to different risk factors related to a much drier local climate, such as hypersaline conditions or drying up of the estuary during the summer months [35], [36].

Our surveys of other tidewater goby in northern California not only indicate that heterogeneity in reproductive timing appears to be severely constrained in some of the remaining populations, but also suggest that such reductions in biocomplexity are often observed in populations with low levels of genetic variation (Figure 3). It should ne noted that narrow size ranges have been detected in populations from the southern portion of the species range, with increased diversification of size composition observed in subsequent surveys [14], but it is unknown how these populations compare to our study populations in terms of genetic diversity and other characteristics. Most populations with restricted size ranges of individuals (and low genetic diversity) included in this study are found along the perimeter of Humboldt Bay, in habitat patches fragmented by draining of wetlands and other human development. Though these populations persist and can reach high abundances, genetic drift following isolation is considered to have eroded genetic diversity, in absence of migration leading to significant degrees of genetic differentiation among populations [16]. The lack of heterogeneity in size composition within these populations indicates that sampled individuals were born within a constrained temporal window, despite the species’ noted ability to reproduce under a wide range of environmental conditions and independent of season [14], [22]. Though respective growth rates may vary among populations, variation in individual size at age is expected to increase (individual fish of the same age may obtain different size at time *t*, Figure S1). Therefore, observed length range can be considered a conservative measure of the extent of the reproductive period (assuming no size selective mortality in the months preceding sampling). Large variation in sizes of tidewater goby is observed in small, isolated habitats when heterozygosity levels are high (for example, VG and PC; Table 1), yet the reproductive period appears constrained when genetic diversity is low, even in large, heterogeneous habitats (SC and LE; Table 1). Observation of this pattern across populations that are reproductively isolated yet in geographic proximity to one another, and therefore subject to nearly identical temperature and precipitation regimes, suggests a correlation between life history variation and genetic diversity in tidewater goby populations.

If reproductive timing in tidewater goby is under genetic influence, the striking asynchrony in reproductive timing in adjacent populations may be explained by the random loss of genetic variation through genetic drift. Though we interpret the results presented herein as suggestive of genetic impoverishment being a causative factor leading to decreased within-population heterogeneity, we recognize that alternative explanations for the observed patter should be considered. Specifically, a reduction in birthdate variation owing to periodic failure in recruitment or size selective mortality due to unsuitable environmental conditions may explain a limited range in the size of individuals and have caused erosion in genetic variation within those populations. However, as recruitment failures occur stochastically and not periodically, we believe that the observed pattern of demographic homogeneity in populations with low genetic diversity, considered in the context of the species’ life history characteristics and their applicability to the dynamic environments in which the tidewater goby occurs, indicates that limited variation is reproductive timing is attributable to reductions in genetic diversity within isolated tidewater goby populations. Irrespective of whether a causative effect exists between genetic- and demographic diversity, limited age variation within these populations renders them particularly susceptible to environmental disturbance during certain times of the year through elimination of stabilizing effects provided by within-population variability.

We do not propose that near-term extinction of extant tidewater goby populations is accurately predicted by measured heterozygosity levels, and while stochastic events, likely in the form of natural salinity increases, will probably trigger future localized extinctions of tidewater goby in the species’ northern range, we believe that this study clearly illustrates that demographically complex populations of this annual species are more resilient to such stressors. Likewise, though environmental factors doubtlessly have some influence on observed length ranges within isolated populations, the highly significant relationship between this measure of life history- and genetic diversity implies that the level of genetic variation is related to demographic heterogeneity. Though a causative relationship may be subject to debate, at a minimum, our results illustrate that a lack of genetic diversity works synergistically with environmental disturbance to influence population extirpation. As such, these results not only affirm the notion that genotypic and phenotypic diversity are tightly linked and deserve equal consideration in the quest for species conservation, but also illustrate the applicability of buffering portfolio effects to isolated populations, which contribute to their resilience against stochastic disturbances.

Our findings suggest that environmental instability during and shortly after periods of peak reproductive activity is of particular concern to the conservation of tidewater goby populations, especially those characterized by low levels of age- and genetic diversity. As a consequence, artificial breaching of estuaries during spring and summer (occasionally practiced for reasons such as draining pasture land inundated by high water levels in the estuary) should be avoided when possible. In addition, cohort assessment (analysis of size/age structure and relative abundance) should be incorporated in future surveys of tidewater goby populations, as this may permit identification of goby populations at elevated risk of near-term extirpation. Lastly, the relationship between genetic diversity and observed age variation should be investigated further, for example by experimental supplementation of genetic diversity in isolated populations through transfers of individuals from nearby, genetically diverse populations. Subsequently, frequent demographic assessments could be used then to evaluate whether reproductive variability has increased as a result of genetic supplementation, which may then receive consideration as a management tool in the quest for conservation of this endangered species.

## Supporting Information

Figure S1 **Schnute’s (1981) four-parameter growth curves for populations of tidewater goby from Big Lagoon (BL), CA, and the Arcata Marsh (AM), CA.**
(TIFF)

Figure S2 **Length frequency composition of tidewater goby from Big Lagoon, CA, sampled monthly between April 2009 and August 2010.**
(TIFF)

Figure S3 **Length frequency histogram of the Big Lagoon, CA, and Arcata Marsh, CA, tidewater goby populations prior and subsequent to a stochastic, temporary increase in salinity.**
(TIFF)

File S1 **Multilocus genotypes for tidewater goby,** ***Eucyclogobius newberryi*** **, from 17 populations in northern California.** Information on relevant primers and amplification protocol can be found in the main text.
(TXT)
