# Supplementary figures and images for: Increased Extinction Potential of Insular Fish Populations with Reduced Life History Variation and Low Genetic Diversity
Source: PLoS One. 2014 Nov 19;9(11):e113139. doi: 10.1371/journal.pone.0113139 (PMC4237396; doi:10.1371/journal.pone.0113139)

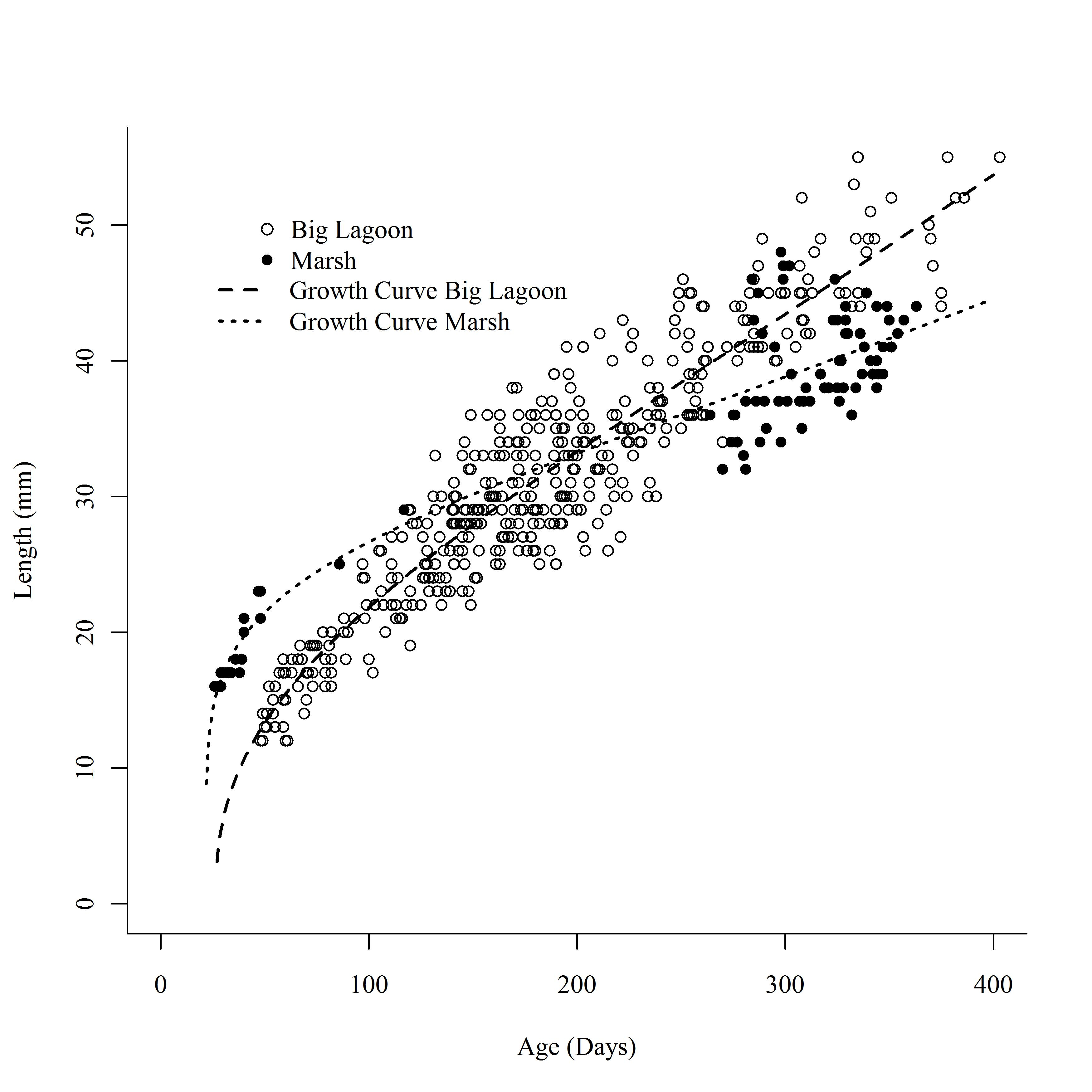

Supplement: Figure S1 — Schnute’s (1981) four-parameter growth curves for populations of tidewater goby from Big Lagoon (BL), CA, and the Arcata Marsh (AM), CA. (TIFF) [file pone.0113139.s001.tiff]

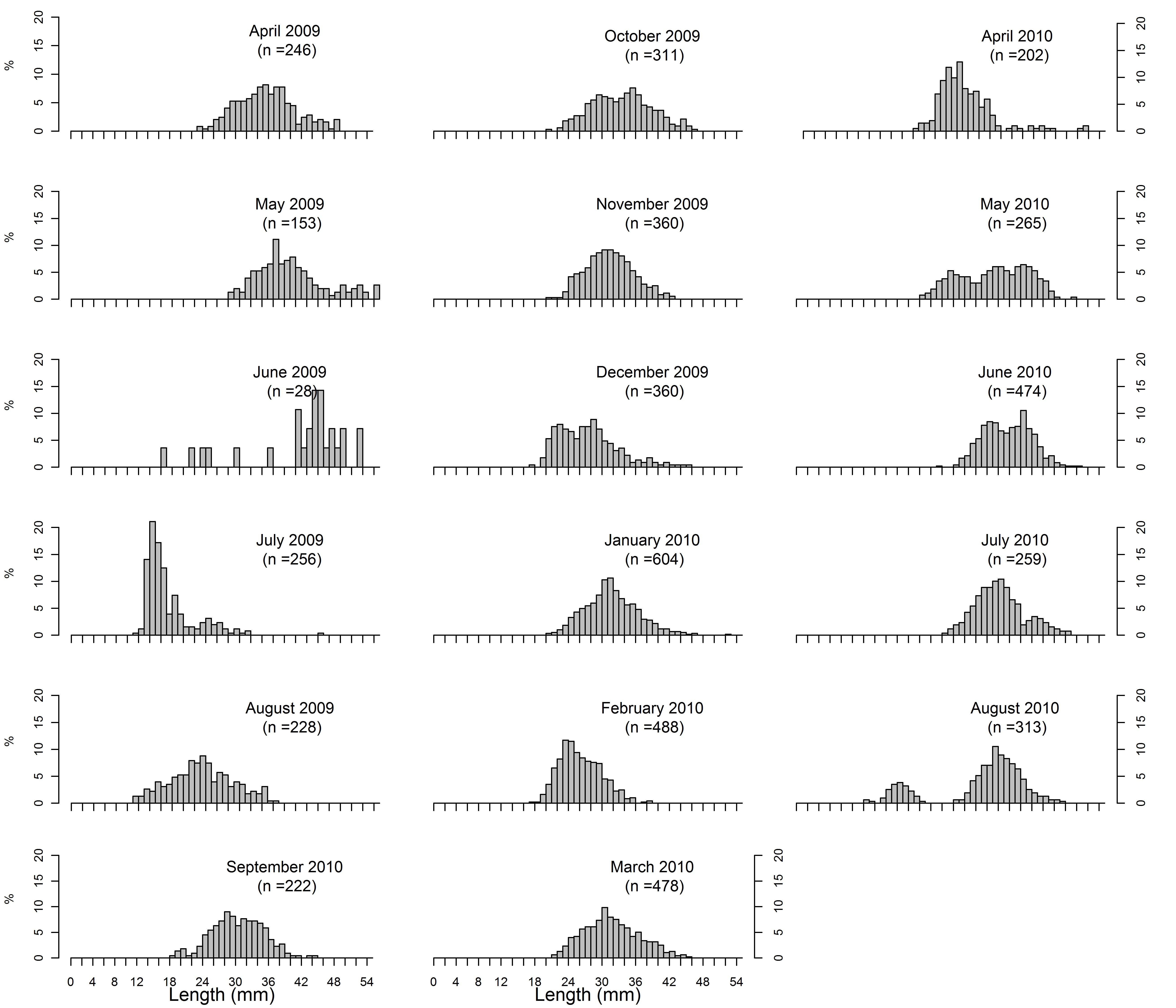

Supplement: Figure S2 — Length frequency composition of tidewater goby from Big Lagoon, CA, sampled monthly between April 2009 and August 2010. (TIFF) [file pone.0113139.s002.tiff]

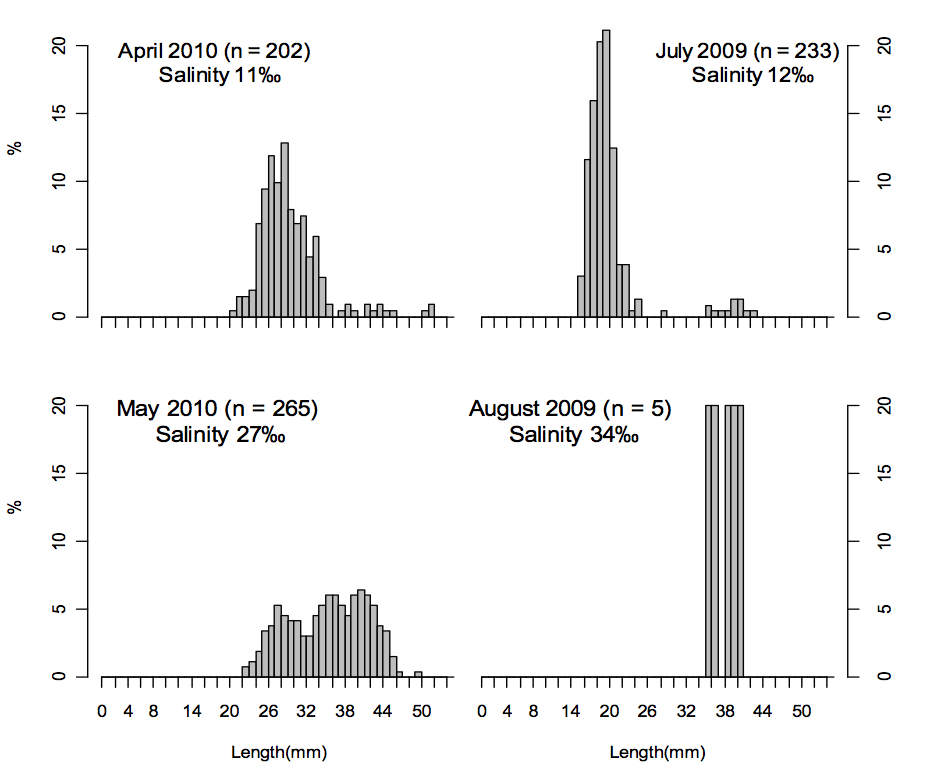

Supplement: Figure S3 — Length frequency histogram of the Big Lagoon, CA, and Arcata Marsh, CA, tidewater goby populations prior and subsequent to a stochastic, temporary increase in salinity. (TIFF) [file pone.0113139.s003.tiff]
